# Retrospective Characterization of Initial Peste des petits ruminants Outbreaks (2008–2012) in the Democratic Republic of the Congo

**DOI:** 10.3390/v13122373

**Published:** 2021-11-26

**Authors:** Leopold K. Mulumba-Mfumu, Mana Mahapatra, Adama Diallo, Brian Clarke, Augustin Twabela, Jean Pierre Matondo-Lusala, Felix Njeumi, Satya Parida

**Affiliations:** 1 Clinical Sciences Department, Faculty of Veterinary Medicine, University of Kinshasa, Kinshasa 1, Democratic Republic of the Congo; leopoldmulumbamfumu@gmail.com; 2 The Pirbright Institute, Ash Road, Pirbright, Woking, Surrey GU24ONF, UK; mana.mahapatra@pirbright.ac.uk (M.M.); bdclarke@protonmail.com (B.C.); 3 UMR CIRAD INRA, Animal, Santé, Territoires, Risques et Ecosystèmes (ASTRE), CEDEX 05, 34398 Montpellier, France; adama.diallo@cirad.fr; 4 Laboratoire National d’Elevage et de Recherches Vétérinaires, Institut Sénégalais de Recherches Agricoles (ISRA), Dakar-Hann, Dakar BP 2057, Senegal; 5 Central Veterinary Laboratory, Gombe, Kinshasa, P.O. Box 8842, Kinshasa 1, Democratic Republic of the Congo; ttaugusha@yahoo.fr (A.T.); lusmatondo2018@gmail.com (J.P.M.-L.); 6 Food and Agriculture Organization of the United Nations (FAO), Viale delle Terme di Caracalla, 00153 Rome, Italy; felix.njeumi@fao.org

**Keywords:** Peste des petits ruminants virus, lineages, PPRV nucleic acid, full genome, PPR in DRC

## Abstract

Peste des petits ruminants (PPR) is an acute, contagious viral disease of small ruminants, goats and sheep. The Democratic Republic of the Congo (DRC) was a PPR-free country until 2007, although in 2006, scare alerts were received from the east and the southwest of the country, reporting repeated mortalities, specifically in goats. In 2008, PPR outbreaks were seen in several villages in the west, leading to structured veterinary field operations. Blood, swabs and pathological specimens consisting of tissues from lungs, spleens, lymph nodes, kidneys, livers and hearts were ethically collected from clinically infected and/or dead animals, as appropriate, in 35 districts. Epidemiological information relating to major risk factors and socio-economic impact was progressively collected, revealing the deaths of 744,527 goats, which converted to a trade value of USD 35,674,600. Samples from infected and dead animals were routinely analyzed by the Central Veterinary Laboratory at Kinshasa for diagnosis, and after official declaration of PPR outbreaks by the FAO in July 2012, selected tissue samples were sent to The Pirbright Institute, United Kingdom, for genotyping. As a result of surveys undertaken between 2008 and 2012, PPR virus (PPRV)-specific antibodies were detected in 25 locations out of 33 tested (75.7%); PPRV nucleic acid was detected in 25 locations out of 35 (71.4%); and a typical clinical picture of PPR was observed in 23 locations out of 35 (65.7%). Analysis of the partial and full genome sequences of PPR viruses (PPRVs) obtained from lymphoid tissues of dead goats collected in Tshela in the DRC in 2012 confirmed the circulation of lineage IV PPRV, showing the highest homology (99.6−100%) with the viruses circulating in the neighboring countries of Gabon, in the Aboumi outbreak in 2011, and Nigeria (99.3% homology) in 2013, although recent outbreaks in 2016 and 2018 in the western part of the DRC that borders with East Africa demonstrated circulation of lineage II and lineage III PPRV.

## 1. Introduction

**Figure 1:**
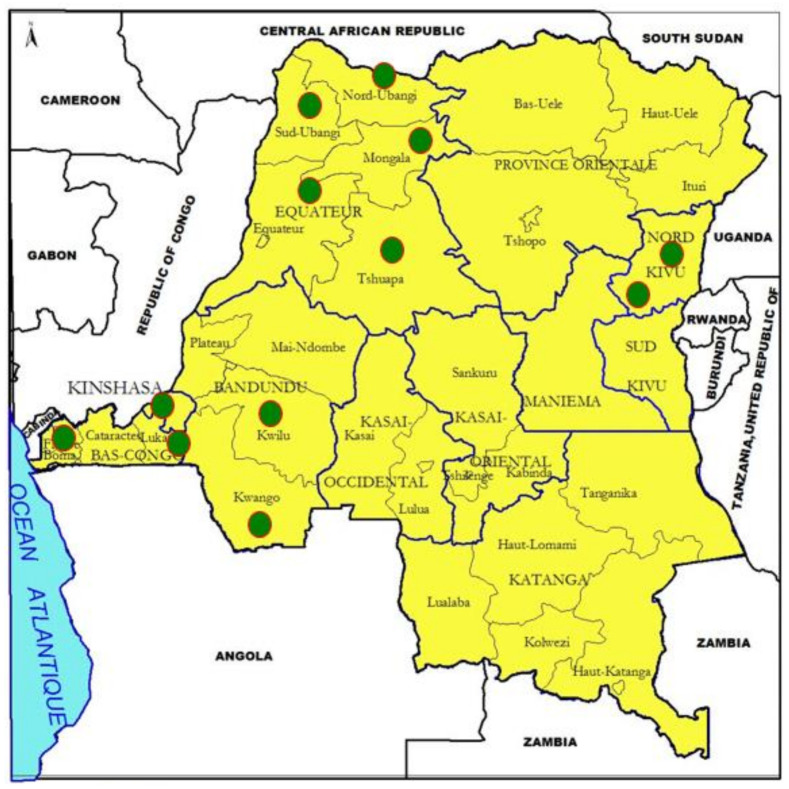
Map of the Democratic Republic of the Congo (DRC). Study areas are represented as green circles.

Peste des petits ruminants (PPR) is a notifiable viral animal transboundary disease, highly contagious in domestic and wild small ruminants [1]. In some geographical regions across the world, PPR is identified under other synonyms such as “Kata, syndrome of stomatitis-pneumoenteritis, ovine rinderpest” [2], and “goat plague, plague of small ruminants” [3]. PPR virus (PPRV) is classified as a member of the morbillivirus genus, in the paramyxovirinae sub-family, paramyxoviridae family and mononegavirales order [2,3]. PPR virus (PPRV), recently named as small ruminant morbillivirus, is mainly circulating in Asia, including Southeast Asia, in Thailand (first outbreaks seen in 2021), the Middle East and all over Africa except a few Southern African countries. PPRV exists as a single serotype with four genetically distinct lineages (I–IV); all four lineages are prevalent in Africa. PPR is a major constraint to international trade due to drastic embargoes forbidding ships to enter or leave ports [4]. It is a threat to food security [5,6] and a bottleneck to rural economies mainly in developing countries where goat and sheep farming is a source of cash income. In developing countries, goats or sheep are called “cows of poor people”, and these are generally women and children [7]. PPR was observed for the first time by Beaton in Nigeria in 1930, but its first description was established in Cote d’Ivoire in 1942 by Gargadennec and Lalane [8]. Morbidity and mortality rates of PPR can reach up to 90−100% in naïve populations [9], and 20−80% in endemic areas [10]. The Democratic Republic of the Congo (DRC) was PPR-free until 2007 when the first outbreaks based on alerts of sporadic cases in Bas Congo state were reported; however, in 2006, scare alerts were received from the southeast and the southwest of the country reporting repeated mortalities, specifically in goats, followed by reports of severe clinical disease in 2008 [11]. The DRC, the second largest country of the African continent (after Algeria), has a total area of 2,345,409 square kilometers (km^2^), with a national 9165 Kms long and porous border shared with nine neighbors, namely, the Republic of Congo-Brazzaville, the Republic of Central Africa, Sudan, Uganda, Rwanda, Burundi, Tanzania, Zambia and Angola (Figure 1). The DRC harbors huge, rainy equatorial forests representing an area of 135.2 million Km^2^, in addition to eight national parks and several nature reserves containing diverse wild small ruminants. Small ruminant husbandry is more exploited in rural than urban areas, and it is familial and mostly practiced by women, as in other regions and countries [4], and young persons, with goats raised more than sheep. Economically, the DRC belongs to the Southern African Development Community (SADC) which is currently at high risk of economic losses [4,11,12]. PPR has currently reached more than half of the total number of territories in the DRC, becoming increasingly endemic. Although the circulation of PPRV was confirmed in huge outbreaks during 2012, reports on the molecular characterization of PPRV from these initial outbreaks (2008−2012) are not yet available. In 2019, Tshilenge and colleagues reported the circulation of lineage II and III viruses in outbreaks (2016 and 2018) in the western part of the country bordering East African countries [13]. The main aim of this retrospective study was to identify the lineage of PPRV that caused the initial devastating outbreaks in the DRC and, also to establish the linkage of this lineage of PPRV with the circulating viruses in its neighboring countries so that the associated risk factors and losses can be measured. Further, assessment of associated risk factors will be helpful for the development and implementation of targeted control strategies to prevent the introduction of new diseases in free countries [12] or disease-free regions in a large country such as the DRC.

## 2. Materials and Methods

### 2.1. Study Areas

This study was focused only on the areas where PPR outbreaks were observed for the first time in the DRC in 2008−2012 (Supplementary Table S1). Currently, the country is divided into 26 administrative provinces; however, at the time of the initial outbreaks, the country had 11 provinces. Most of the former provinces were transformed into new provinces according to the new administrative law. The new provinces are further divided into territories, with a total of 145 territories in the DRC. Furthermore, territories are divided into sectors. The territories, sectors and villages covered by this study belong to 5 former provinces out of 11, as indicated on the map (Figure 1). These outbreak locations were Lukunga, Funa, Tshiangu, Matete, Kinshasa-commune and Mont-Ngafula from Kinshasa City Province; Masisi and Walikale from Nord-Kivu Province; Matadi, Tshela, Lukaya and Madimba from the former province of Bas Congo (nowadays Kongo Central); Bulungu, Kikwit, Kwilu, Kwango and Masimanimba from the former province of Bandundu (nowadays divided into 3 new provinces); and Tshuapa, Yakuma, Gbadolite, Gemena and Lisala from the former province of Equateur (nowadays divided into 4 new provinces) (Figure 1). It appears that most of the surveyed areas were situated in the western part of the country. During the field operations, the study areas were visited directly by local private and/or official veterinarians and veterinary scientists.

### 2.2. Ethical Statement

Samples were collected for disease diagnosis under routine veterinary service work in the DRC; no permits were required for collection of samples. Selected samples were sent to The Pirbright Institute, United Kingdom, for further diagnosis and virus genotyping. When consulted, the local Pirbright animal welfare ethical review board (AWERB) confirmed that no further approval was necessary as the samples were collected primarily for veterinary diagnosis purposes in the DRC, and not for research.

### 2.3. Sampling

During field operations, some outbreaks with PPR pathognomonic signs were still ongoing, and this was the case for most of the sites. Whole blood samples as well as ocular, nasal and rectal swabs were collected from the suspected animals. Tissue pieces from spleens, lungs, livers, hearts and lymph nodes were collected from freshly dead animals during postmortem examination. These samples were collected for routine diagnostic work for confirmation of alerts. Zipper-top poly-plastic bags (SIGMA-ALDRICH, St Louis, MO, USA) were used to store tissue samples for RT-PCR. Blood samples were collected in vacutainer tubes. The samples were placed on ice in 20 L field boxes. All boxes were transported to the national reference laboratory at Kinshasa for routine diagnostic testing. Once in the laboratory, serum was separated from blood and stored at −20 °C, and the remaining samples were stored at −80 °C until use.

### 2.4. Clinical and Postmortem Examination

The procedures classically recommended for clinical examination of herds and individual animals in outbreak situations [14] were strictly followed. The main clinical exploration procedure for this approach was physical inspection of suspected animals. Rectal body temperature was recorded using a thermometer. Only one or two suspected animals per flock were examined to confirm pyrexia to corroborate other clinical signs. Pathological gross changes were observed through external examination of dead animals’ carcasses as well as during internal examination. Necropsy and clinical data per outbreak were recorded. 

### 2.5. Antibody/Antigen Detection

PPRV-specific antibodies were detected using a recombinant N protein based cELISA Kit [15,16] supplied by IDvet, France. The ELISA was performed following the manufacturer’s instructions. Immuno-histochemical (IHC) staining was performed in some paraffin-embedded tissues to confirm PPR disease [17]. 

### 2.6. RNA Extraction, Reverse Transcription-Polymerase Chain Reaction (RT-PCR) and Sequencing

Two different laboratories were involved in these analyses: (1) the Central Veterinary Laboratory (CVL) at Kinshasa that confirmed the outbreaks, and (2) The Pirbright Institute, UK, that carried out advanced analyses for virus genotyping. At the CVL, total RNA was extracted directly from swabs and homogenized tissues using RNeasy Mini Kit (Qiagen, Germany). RT-PCR reactions were performed using One Step RT-PCR kit (Qiagen) with the primer set NP3 (5′ TCTCGGAAATCGCCTCACAGACTG 3′) and NP4 (5′ CCTCCTCCTGGTCCTCCAGAATCT 3′) for the amplification of the partial N gene with thermocycling conditions as previously described [18]. At The Pirbright Institute, total RNA was extracted from the homogenized tissue samples using Trizol^TM^ (Invitrogen) as per the manufacturer’s instructions. The viral RNA was reverse transcribed, and the C-terminus of the N gene was amplified using the primer pair NP3/NP4 and superscript III one-step synthesis kit (Invitrogen, Carlsbad, CA, USA) [18]. For full-length genome sequencing, the viral RNA was reverse transcribed using superscript III first strand synthesis kit (Invitrogen). For amplification of genome fragments, a hemi-nested RT-PCR was carried out as described previously [19,20,21]. Amplification of the terminal 5′ and 3′ ends of the PPRV genome was accomplished by rapid amplification of cDNA ends (RACE), as previously described [21]. The PCR amplicons were purified using the GE Healthcare Illustra GFXPCR purification kit (GE Healthcare, Pittsburgh, PA, USA) according to the manufacturer’s instructions and sequenced using BigDye1 Terminator v3.1 Cycle Sequencing Kit (Applied Biosystems, Carlsbad, CA, USA) on an ABI 3730 machine. Sequences were assembled and analyzed using SeqMan pro (DNAStar Lasergene 13.0). Nucleotide sequences of the viruses were aligned using the CLUSTAL X multiple sequence alignment program [22] or MUSCLE [23].

### 2.7. Sequence Datasets

Forty-three complete PPRV genome sequences were retrieved from GenBank on 12 March 2021. In addition, representative partial N sequences (*n* = 82) from around the world were included in the analysis. Sequences obtained from live attenuated vaccine strains Sungri/96 (KJ867542, KF727981) and Nigeria75/1 (X74443, HQ197753) were excluded prior to analysis as these sequences have previously been shown to substantially skew phylogenetic analyses [20,21,22,23]. Additionally, the two DRC sequences generated in this study were included in the analysis either as full-length sequences or as partial N gene sequences, making a total of 44 full genome and 84 partial N gene sequences.

### 2.8. Risk Factor Assessment

As demonstrated in other studies, assessment of associated risk factors [24] is helpful for the development and implementation of targeted control strategies [12] to prevent the introduction of a virus to disease-free countries [4] or, if already introduced, to control its spread to new areas. The first cases of PPR in the DRC were identified in some western provinces of the country in 2008. For a better understanding of the disease occurrence, it was decided to question stakeholders in order to prepare a structured surveillance protocol that may be appropriate for new introductions of PPRV, specifically when countries face challenges for the first time by the incursion of an exotic disease. Oral queries were made using a participatory procedure by interviewing animal owners, local veterinary officers, private veterinarians, para-veterinarians and local FAO staff as recommended by OIE [10]. Similar outputs have been established with structured SWOT analysis using the FAO Surveillance Evaluation Tool (FAO-SET). The score is 0 if the risk is non-existent, and 1 if there is a potential risk. Crucial risk factors associated with the DRC epidemiological profile are listed in Table 1.

### 2.9. Estimation of Mortalities

Collection of initial outbreak loss data was started when PPR was spreading across the country, and it was progressively completed as an increasing number of districts were affected. Exhaustive data covering a two-year time period (2010−2012) were selected for detailed analysis. The operation was carried out in a participatory way, with data provided directly by animal owners, local veterinarians and para-veterinarians, as recommended by OIE [10]. Provincial FAO representatives were also consulted. The operation became nationwide as more areas were invaded by the spread of the disease, but as for other commodities, the prices of animals including their meat were fixed in provinces, which meant they were not the same and based on provinces’ realities. Following the advice of animal health economists, the operation was altered to be more structured with regard to quantification of financial losses caused by animal diseases [25]. The survey covered a total of sixty outbreaks, not exhaustively, and only accessible territories and important urban municipalities with qualified human resources were included. 

## 3. Results

### 3.1. Affected Areas and PPR Clinical Profile

Based on initial alerts associated with PPR-like disease received from the breeders, the local official and private veterinarians visited a total of 35 locations/districts (*n* = 35) representing 5 provinces out of the 11 old provinces. Regarding the origin of alerts, the majority of the visited locations were situated in the west of the DRC (Table 1 and Figure 1). PPR occurrence was established in 27 locations either by observation of clinical signs or laboratory tests. Of these 27 occurrences, 23 (76.64%) were recorded as severe clinical outbreaks involving severe typical clinical signs of PPR, abrupt death without symptoms, a high mortality rate and pathognomonic clinical signs. These 23 clinical outbreaks were confirmed by detection of PPRV RNA in RT-PCR, and 21 (91%) out of 23 were confirmed by detection of PPRV-specific antibodies in cELISA (Supplementary Table S1). Although PPR-specific clinical signs were not very evident in the remaining four suspected locations, all four were positive for PPRV-specific serum antibodies; however, only two of them were positive for the presence of PPRV RNA. Based on a temporo-spatial approach, it was observed that 4 provinces (80%) out of the 5 inspected were affected by PPRV, and at the country level, 4 provinces out of 11 (about 36 %) were affected during the study period. Finally, the DRC was officially declared as endemic to PPR on July 7th, 2012 [11]. 

With respect to the farming system, the majority of the severe outbreaks observed were associated with the backyard system; these are familial and half-traditional systems where animals are clustered behind habitations at night in order to prevent thefts and released during the day for feeding by scavenging and free ranging [6] across the villages. Only two commercial farms reported severe PPR outbreaks in peri-urban areas, which is relatively low compared to the number of outbreaks observed in backyard systems. In terms of seasons, only two epidemiological events occurred at the end of the dry season; the rest of the cases were in the rainy season, as has been described [15] for humid and sub-humid tropics such as the DRC, but the two dry season outbreaks continued into the rainy season. 

The most observed clinical signs in surveyed areas were pathognomonic for PPR; these were nasal and ocular muco-purulent discharges, ulcerative stomatitis, necrotic rhinitis, ecthyma, mouth swelling and diarrhea, in addition to prodromal and general manifestations such as pyrexia over 40 °C, depression, anorexia, lameness, respiratory distress and peracute disease marked by abrupt death of apparently healthy animals that were symptomless. 

### 3.2. Antibody Detection

The serum samples collected from locations suspected of PPR or PPR-like epidemiological events (*n* = 33) were only tested. PPRV-specific antibodies were detected in serum samples originating from 25 locations of the 33 (75.7%) tested (Supplementary Table S1). These results correlate with the RT- PCR result (25/35 or 71.4%). In general, the RT-PCR results were largely supported by serology and clinical signs. 

### 3.3. Reverse Transcription-Polymerase Chain Reaction (RT-PCR)

PPRV nucleic acids were detected in 25 locations out of the 35 (71.4%) that reported alerts (Supplementary Table 1). Nasal and ocular swabs in addition to tissue specimens revealed positive results when routinely tested by RT-PCR using primer sets located in the N protein gene, which is the major viral protein produced in the highest amount in Morbilliviruses, and most recommended for geographical clustering of lineages due to its high level of resolution [26].

### 3.4. Sequencing Outputs

**Figure 2:**
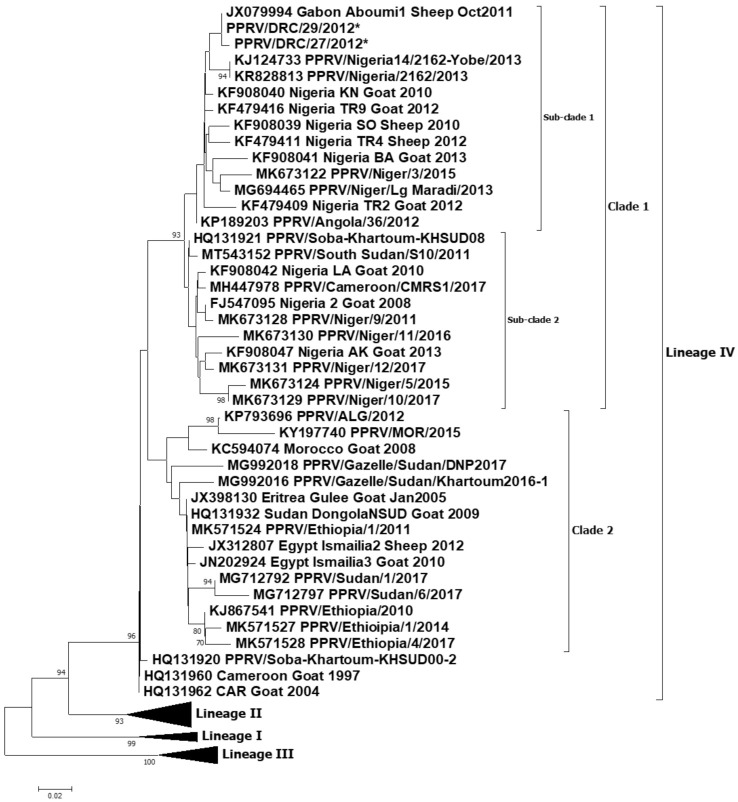
Neighbor-joining tree constructed using partial N gene sequences of peste des petits ruminants virus (PPRV), showing relationships among the PPRV lineage IV isolates circulating in Africa. The Kimura 2-parameter model was used to calculate percentages (indicated by numbers beside branches) of replicate trees in which the associated taxa clustered together in 1000 bootstrap replicates. The sequences from the DRC generated in this study (accession numbers: PPRV/DRC/Tshela/27/2012-OL310685 and PPRV/DRC/Tshela/29/2012-OL310686) are indicated by a star. Scale bar indicates nucleotide substitutions per site.

**Figure 3:**
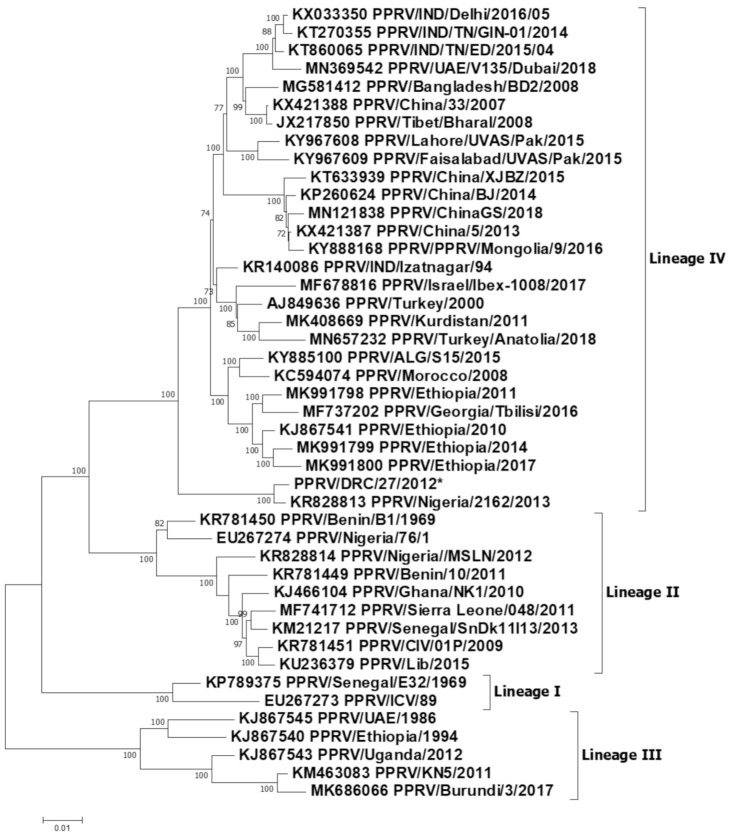
Neighbor-joining tree constructed using full genome sequences of peste des petits ruminants virus (PPRV), showing relationships among the PPRV isolates. The Kimura 2-parameter model was used to calculate percentages (indicated by numbers beside branches) of replicate trees in which the associated taxa clustered together in 1000 bootstrap replicates. The sequence from the DRC generated in this study (accession number PPRV/DRC/Tshela/27/2012-OL310685) is indicated by a star. Scale bar indicates nucleotide substitutions per site.

A total of one partial N gene sequence and one full genome sequence of PPRV were generated in this study. These samples originated from the PPR outbreak in Tshela (Bas Congo province) in March 2012 and have the following accession numbers: PPRV/DRC/Tshela/27/2012-OL310685 and PPRV/DRC/Tshela/29/2012-OL310686. For the partial N gene dataset, the relevant part of the N gene from the full genome generated in this study was extracted for subsequent analysis. The two partial N gene sequences were identical, except for a non-synonymous change (A-G) at position 1428 of the N gene open reading frame (ORF). A phylogenetic analysis of these sequences revealed that the viruses causing PPR outbreaks in 2012 in the DRC belong to lineage IV (Figure 2 and Figure 3). All the lineage IV PPRVs circulating in Africa can be divided into two clades (Figure 2): one belongs to the West and Central African clade, and the second belongs to the East and North African clade. The DRC viruses grouped together with the West and Central African viruses from Nigeria, Niger, Gabon and Angola. Further homology analysis revealed the PPRV/DRC/27/2012 full genome to be 99.30% homologous with the full genome of the PPRV/Nigeria/2162/2013 virus (accession no. KR828813). Similarly, analysis of partial N gene homology revealed 99.6−100% homology of the PPRV/DRC/29/2012 and PPRV/DRC/27/2012 viruses with the Gabon/2011 partial N gene (accession no. JX079994), and 98.40% homology with the PPRV/Nigeria/2162/2013 virus (accession no. KR828813).

### 3.5. Risk Factor Assessment

The identified risks factors were lack of public awareness [10] in terms of knowledge, attitude and practice; direct contact with other neighboring countries through the porous national borders [9]; interactions between wildlife and livestock [9,24], and farming systems such as familial backyards of small stock clustered behind the gardens at night to prevent theft but released during the day for feeding by free ranging; political crises [9,10] ending in forced movements of human populations with their livestock; poor biosafety measures at the farming level; increase in uncontrolled animal movements including transhumance and nomadism in some DRC geographical zones; climate change [12] ending in drought, flooding of grasslands and perversion of agro-pastoral seasons; lack of riposte in real time during outbreak occurrence; lack of quarantine infrastructures on the national borders; deforestation ending in destruction of insects’ habitats which finally invade humans’ environments [8]; transfrontier markets and commercial transactions such as those of Zongo, Lufu, Pweto, Tshela and Moanda; lack of systematic vaccination campaigns; lack of surveillance; separated fences in cross-border nature reserves; and poor communication and reporting capacity (Table 1). Upon the assessment, lack of public awareness (especially lack of knowledge, attitude and practice) was evident. The identified risk factors might have contributed to the spread of PPR in the DRC: indeed, the disease was identified only in the west of the country (Figure 1), but it has now spread and become endemic in the majority of the 11 provinces that are at high risk. Provinces showing the highest number of risk factors were categorized as very high risk (the most exposed), with endemic PPR and presumably symptomless susceptible hosts species. The others were ranked in two subdivisions as follows: high risk and low risk/currently disease free, which require specific control options in regard to the OIE/FAO global PPR eradication strategies [12].

### 3.6. Estimate of Mortalities

PPR is a poverty-inducing disease affecting food and nutritional insecurity, touching vulnerable populations, especially in rural areas [5,8]. Important data dealing with heavy mortalities were collected in several outbreak areas. For financial reasons, only accessible locations and those with qualified human resources (*n* = 60) were surveyed in 2010 and 2011 (Supplementary Table S2). This structured account revealed colossal losses of 744,527 goats killed by PPR, which, when converted into cash income, translated to a value of USD 35,674,600(Supplementary Table S2); when temporal-spatially converted at constant average prices in a particular base year, this translated to USD 32,507,483 and USD 2,401,569, respectively, for 2010 and 2011.

## 4. Discussion

The DRC was one of the OIE member states free of PPR until 2007, where the first outbreak was experienced in the southeast part of the country in 2008 [11]. As reported in the Results section of this article, partial and full genome sequences of PPRV from 2012 outbreaks were analyzed by two phylograms: one based on virus full genome sequences and the second based on partial sequences of the N protein gene. Both the full genome and partial gene neighbor-joining trees confirmed the outbreaks were caused by PPRV lineage IV. The genetic relationship, as elicited by the full genome sequencing, demonstrated homology (99.30%) between the PPRV involved in the Tshela outbreak in March 2012 and Nigeria KR828813_2013, while the partial N gene sequencing revealed homology (99.60−100%) between the Tshela outbreak virus and the Gabon/Aboumi 1 Sheep October 2011 virus. According to previous sequence analysis, the Aboumi outbreak virus was genetically close to the virus involved in PPR outbreaks in Cameroon [27]; considered together, these results elicit a sub-Saharan African diffusion, as detailed below (Table 2). However, in our study, partial N gene sequences (full genome not available) for both the available Cameroon outbreak viruses in 2004 and 2017 revealed 95.2% and 96.8% homology, respectively, with the partial N gene sequences of the DRC 2012 viruses.

Tshela in Bas Congo (5°20′S/13°16′E) is located on the edge of the national terrestrial border between the DRC and Congo-Brazzaville, which is not too far from Aboumi village in Gabon (Figure 1) [27]. According to previous reports, lineage IV viruses were considered as being confined to the Middle East, the Indian sub-continent and Southeast Asian countries including China and Mongolia. Based on recent results for Central Africa, lineage IV was reported in the October 2011 outbreak in the southeast of Gabon, and it was genetically close to PPRV strains circulating in Cameroon [27]. Unfortunately, the full genome sequence of the Gabon virus is not available in the NCBI database. Further, phylogenetic analysis revealed that lineage IV viruses from Gabon as well as the DRC grouped together with viruses from Nigeria, Niger and Angola (Figure 2, sub-clade 1). The sequence homology of lineage IV PPRVs from other neighboring countries such as Cameroon, CAR, South Sudan and Uganda revealed less similarity with the lineage IV viruses from the DRC. Cross-border transmissions between the DRC and Gabon might be incriminated, given the frequent illegal movements and commercial transactions in these areas that are traditionally shared by close tribes. At the continental level, it is established that all four lineages of PPRV have been circulating in Africa for several years [9,28]. Therefore, based on the data generated in this study and other previous reports [13], the DRC is currently harboring three lineages of PPRV: II and III (2016−2018 outbreaks, reported in 2019), and IV (this study) (Table 2). PPRV lineage IV is also largely distributed in the majority of the immediate neighboring countries [27,29] (Table 2). The rapid spread of PPRV across a 2,345,409 km^2^ country within a very short period of time might be associated with the wide range of risk factors pointed out and assessed within this study (Table 1). The induced heavy mortality losses could also be considered to contribute to the persistence of PPR; as a matter of fact, during outbreaks and field operations, it was observed that commercialization of sick animals was increasing. This is another type of risk which can not only contribute to the spread of the disease but could also contribute to the maintenance of strains and other dormant outbreaks. Methodic assessment of PPR exposure through structured procedures such as scoring and highlighting critical risk factors, depending on countries, should be encouraged. As an example, Orientale Province is one of the hot spots for many transboundary diseases including zoonotic diseases. In this study, Orientale Province scored the highest, with a total risk cartography score of 18, while the provinces of Kasai Oriental, Maniema and Kinshasa appeared to be safer, with lower scores (Table 1). Therefore, risk factor analysis is helpful to prevent the spread of the disease to new areas. It should also enable cost-effective and targeted control strategies for efficient and effective eradication. The wildlife component of risk factors should be regularly surveyed in the DRC due to several ecological scenarios dominated by the huge equatorial forest which covers 56% of the country [30], harboring a huge biodiversity of receptive animal species. Beside the equatorial forest, there is also the huge Mayumbe forest within the biosphere of Luki, starting from the Bas-Fleuve District, linking Gabon through Congo-Brazzaville. Tshela and Aboumi belong to this same ecosystem. Finally, there is the huge oceanic forest massif of mangroves which borders the left littoral of the Atlantic Ocean, which begins at the mouth of the Congo River and ends at the Gulf of Guinea through the Littoral Region of Cameroon. The DRC has eight national parks, of which two are very large: (1) Caramba is shared with South Sudan in the northeast, and (2) Virunga is shared with Rwanda and Uganda in the east; there are other nature reserves such as the large one shared with Zambia in the north, which is a national park on the other side. These ecosystems harbor several interfaces where PPR-susceptible small ruminant species share the same feeding grasslands and water spaces which presents a high risk of PPR spillover from one species to others, as already demonstrated by an earlier report [1].

On the other hand, severe PPR forms with a peracute to an acute course in naïve populations in new areas could be due to the lack of immunity, given the fact that the small ruminant populations of the DRC are facing a primo-infection episode, which might be the cause of the dramatic clinical profile with the highest morbi-mortality rates. Other associated factors relating to the huge mortality rates might be due to over-infection by another endemic disease present in the DRC, such as Pasteurollosis, and external and internal parasites such as coccidiosis, strongylosis, cysticercosis, cawdriosis, manges, dermatophilosis, contagious ecthyma and caprine contagious peripneumonia, as previously reported by other researchers [3,15]. It has also been reported that Sahelian goats and sheep, given their long period of exposure to PPRV, are resistant to PPR [15]; this might not be the case for the DRC small ruminant population as it is their primo-infection.

With regard to the economic losses, only mortalities were structurally documented; other types of losses such as veterinary drug expenses, veterinarians’ honoraria, disposal of dead animal carcasses and disinfection were not covered. 

In conclusion, this study reveals the importance of cross-border virus movement in causing new outbreaks and the spread of the studied disease to low-risk and disease-free areas. In Saharan and sub-Saharan Africa, in order to make PPR eradication successful, some critical strategies need to be adapted at the regional level. National contingency plans should be updated and regionally harmonized during implementation. Active surveillance and control strategies including mass vaccination campaigns targeting the region need to be coordinated [12]. 

## Figures and Tables

**Table 1:** Risk factor assessment of different provinces of the DRC. A score of 1 indicates there is a potential risk within the area; 0: there is no potential risk within the area. The provinces with a risk cartography score of ≥14 indicates at very high risk (highlighted red); ≥12 and <14: at high risk (highlighted yellow) and ≤11: at low risk or are provinces with several disease-free areas (highlighted green). This is the current picture in the DRC, which might be exploited as a starting point, if a target control strategy is adopted by the government within the SADC space.

FORMER 11 PROVINCES OF THE DRC	Knowledge Attitude and Practice; public awareness	Easy Contact with other Countries (porous border)	Forest Exploitation (Mining, Agriculture, wood industry and hunting)	Interfaces: Wild, Domestic Animals and humans	Farming System (Backyard with animals scavenging during the day.	Political Crisis with Forced movements of populations including small scale breeders	Poor Biosafety System at the farming level	Rapid Increase in Population with low offer and high demand at the national level	Transhumance and Nomadism (East and North East chiefly)	Climate Change Drought and/Floods; seasons	Lack of Riposte in Real Time	Lack of Quarantine Infrastructures at the borders level	Deforestation, Release of Blood sucking vectors	Low Number of Qualified Human resources	Commercial Exchanges through borders markets (cases of Zongo, Lufu, Pweto, Cabinda)	Lack of Systematic Vaccination campaigns programme	Lack of Surveillance and Fences in wildlife reserves (cases of Virunga, Caramba, etc.)	Poor Capacity of Communication and reporting. including for alerts,	Risk Cartography
**Equateur**	**1**	**1**	**1**	**1**	**1**	**1**	**1**	**0**	**1**	**1**	**1**	**1**	**1**	**1**	**1**	**1**	**1**	**1**	**17**
**Bandundu**	**1**	**1**	**1**	**1**	**1**	**0**	**1**	**0**	**0**	**1**	**1**	**1**	**0**	**1**	**1**	**1**	**1**	**1**	**14**
**Bas Congo**	**1**	**1**	**1**	**1**	**1**	**0**	**1**	**0**	**0**	**1**	**1**	**1**	**1**	**1**	**1**	**1**	**1**	**1**	**15**
**Kasaï Or.**	**1**	**0**	**0**	**0**	**1**	**0**	**1**	**1**	**0**	**1**	**1**	**1**	**1**	**1**	**0**	**1**	**1**	**1**	**12**
**Kasaï Occ.**	**1**	**1**	**1**	**1**	**1**	**0**	**1**	**1**	**0**	**1**	**1**	**1**	**1**	**1**	**1**	**1**	**1**	**1**	**16**
**Katanga**	**1**	**1**	**0**	**0**	**1**	**0**	**1**	**1**	**0**	**1**	**1**	**1**	**1**	**0**	**1**	**1**	**1**	**1**	**13**
**Kinshasa**	**1**	**0**	**0**	**0**	**0**	**0**	**1**	**1**	**0**	**1**	**1**	**1**	**1**	**0**	**0**	**1**	**1**	**1**	**10**
**Maniema**	**1**	**0**	**0**	**1**	**1**	**0**	**1**	**0**	**0**	**1**	**1**	**1**	**0**	**1**	**0**	**1**	**1**	**1**	**11**
**North Kivu**	**1**	**1**	**0**	**1**	**1**	**1**	**1**	**1**	**0**	**1**	**1**	**1**	**0**	**0**	**1**	**1**	**1**	**1**	**14**
**Orientale**	**1**	**1**	**1**	**1**	**1**	**1**	**1**	**1**	**1**	**1**	**1**	**1**	**1**	**1**	**1**	**1**	**1**	**1**	**18**
**South Kivu**	**1**	**1**	**0**	**0**	**1**	**1**	**1**	**1**	**0**	**1**	**1**	**1**	**0**	**0**	**1**	**1**	**1**	**1**	**13**
**Risk weight**	**11**	**8**	**5**	**7**	**10**	**4**	**11**	**7**	**2**	**11**	**11**	**11**	**7**	**7**	**8**	**11**	**11**	**11**	

**Table 2:** List of PPRV lineages circulating in the DRC and neighboring countries.

Country	Lineages of PPRV Outbreaks	Year of Outbreak with Cited References
Central African Rep (CAR)	IV	2004 [26]
South Sudan	IV	2011 [26]
Uganda	IV, III	2012 (IV) and 2014 (III) [20,26]
Tanzania	II, III, IV	2008 (seropositive), 2013 (III), 2011 (II and IV) [1,31,32,33]
Angola	IV	2012 [26]
Gabon	IV	2011, 2007 (seropositive) [26,30]
Cameroon	IV	1997, 2017 [26]
Burundi	III	2017 [26]
DRC	II, III, IV	2011−2012 (IV, in this study), 2016−18 (II and III) [13,34]

## Data Availability

Not applicable.

## Supplementary Materials

The following are available online at https://www.mdpi.com/article/10.3390/v13122373/s1, Table S1: title, Initial alerted outbreaks in DRC goats during 2008–2012. Table S2: title, Estimated losses in USD during PPR outbreaks in the DRC (2010–2012)
