# Supplementary material for: Retrospective Characterization of Initial Peste des petits ruminants Outbreaks (2008–2012) in the Democratic Republic of the Congo
Source: Viruses. 2021 Nov 26;13(12):2373. doi: 10.3390/v13122373 (PMC8708707; doi:10.3390/v13122373)
Supplement: Supplementary file 1 [file viruses-13-02373-s001.zip › viruses-1425477suppMerged.pdf]

Table S1. PPR cases in goats - initial alerted outbreaks - chronological list. Tissues consisted of pieces of spleen, lymph nodes, lung, liver, heats and kidney. Swabs were ocular, nasal and rectal.

N°	Date	District	GPS	Province	Season	Farming system	Sample nature	cELISA	RT - PCR	Observations
1	Sept 2008	Lukunga	4°19′S, 15°19′E	Kinshasa	rainy	Back yard	Sera, Swabs, Tissues	negative	negative	No clinical disease
2	Sept 2008	Funa	4°19′S, 15°19′E	Kinshasa	rainy	Back yard	Sera, Swabs, Tissues	negative	negative	No clinical disease
3	Sept 2008	Funa	4°19′S, 15°19′E	Kinshasa	rainy	Back yard	Sera, Swabs, Tissues	positive	negative	No clinical disease
4	Sept 2008	Masisi	23°54′S,28°48′E	Nord Kivu	rainy	commercial farm	Sera, Swabs, Tissues	negative	negative	No clinical disease
5	Sept 2008	Tshiangu	4°23′S,15°58′E	Kinshasa	rainy	commercial farm	Sera, Swabs, Tissues	negative	negative	No clinical disease
6	Sept 2008	Walikale	1°25′S, 28°4′ E	Sud Kivu	rainy	commercial farm	Sera, Swabs, Tissues	negative	negative	No clinical disease
7	Dec 2008	Matadi	5°49′S,13°28′ E	Bas Congo	rainy	Abattoir	Sera, Swabs, Tissues	positive	negative	No clinical disease
8	Dec 2008	Matete	4°19′S, 15°19′E	Kinshasa	rainy	Market	Sera, Swabs, Tissues	negative	negative	No clinical disease
9	Jan 2009	Bulungu	4°32′S,18o36′E	Bandundu	rainy	Back yard	Sera, Swabs, Tissues	negative	negative	No clinical disease
10	Jan 2009	Masimanimba	4°46′S, 17°53′E	Bandundu	rainy	Back yard	Sera, Swabs, Tissues	Positive	positive	No clinical disease
11	Jan 2009	Lukaya	4°35′S, 15°10′ E	Bas Congo	rainy	Back yard	Sera, Swabs, Tissues	Positive	positive	No clinical disease
12	Feb 2009	Kikwit	5°01′S, 18°49′E	Bandundu	rainy	Back yard	Sera, Swabs, Tissues	negative	negative	No clinical disease
13	Apr 2009	Tshuapa	0°44′S, 19°11′E	Equateur	rainy	Back yard	Sera, Swabs, Tissues	positive	positive	Severe clinical disease
14	Jun 2009	Kinshasa	4°19′S, 15°19′E	Kinshasa	rainy	Back yard	Sera, Swabs, Tissues	not done	positive	Severe clinical disease
15	Aug 2009	Tshuapa	0°44′S, 19°11′E	Equateur	rainy	Back yard	Sera, Swabs, Tissues	positive	positive	Severe clinical disease
16	Oct 2009	Tshela	5°20′S,13°16′ E	Bas Congo	rainy	Back yard	Sera, Swabs, Tissues	positive	positive	Severe clinical disease
17	Nov 2009	Kwilu	5°07′S,18°39′ E	Bandundu	rainy	Back yard	Sera, Swabs, Tissues	positive	positiv	Severe clinical disease
18	Nov 2009	Tshuapa	0°44′S, 19°11′E	Equateur	rainy	Back yard	Sera, Swabs, Tissues	positive	positiv	Severe clinical disease
19	May 2010	Tshuapa	0°44′S, 19°11′E	Equateur	rainy	Back yard	Sera, Swabs, Tissues	positive	positive	Severe clinical disease
20	July 2010	Lukunga	4°19′S, 15°19′E	Kinshasa	rainy	Back yard	Sera, Swabs, Tissues	positive	positive	Severe clinical disease
21	Sept 2010	Tshuapa	0°44′S, 19°11′E	Equateur	rainy	Back yard	Sera, Swabs, Tissues	positive	positive	Severe clinical disease
22	Oct 2010	Mont Ngafula	4°25′S,15°17′ E	Kinshasa	rainy	Commercial farm	Sera, Swabs, Tissues	positive	positive	Severe clinical disease
23	Oct 2010	Tshuapa	0°44′S, 19°11′E	Equateur	rainy	Back yard	Sera, Swabs, Tissues	positive	positive	Severe clinical disease
24	Nov 2010	Mont Ngafula	4°25′S,15°17′ E	Kinshasa	rainy	Commercial farm	Sera, Swabs, Tissues	not done	positive	Severe clinical disease
25	Jan 2011	Madimba	4°59′S, 15°07′E	Bas Congo	rainy	Back yard	Sera, Swabs, Tissues	positive	positive	Severe clinical disease
26	Feb 2011	Tshuapa	0°44′S, 19°11′E	Equateur	dry	Back yard	Sera, Swabs, Tissues	positive	positive	Severe clinical disease
27	Mar 2011	Tshuapa	0°44′S, 19°11′E	Equateur	dry	Back yard	Sera, Swabs, Tissues	positive	positive	Severe clinical disease
28	Apr 2011	Tshuapa	0°44′S, 19°11′E	Equateur	rainy	Back yard	Sera, Swabs, Tissues	positive	positive	Severe clinical disease

29	May 2011	Tshuapa	0°44′S, 19°11′E	Equateur	rainy	Back yard	Sera, Swabs, Tissues	positive	positive	Severe clinical disease
30	July 2011	Yakuma	4°05′N, 22°26′E	Equateur	rainy	Back yard	Sera, Swabs, Tissues	positive	positiv	Severe clinical disease
31	Sept 2011	Lisala	4°05′N, 21°30′E	Equateur	rainy	Back yard	Sera, Swabs, Tissues	positive	positiv	Severe clinical disease
32	Sept 2011	Gbadolite	4°17′N, 21°37′E	Equateur	rainy	Back yard	Sera, Swabs, Tissues	positive	positive	Severe clinical disease
33	Sept 2011	Gemena	3°15′N,19°46′ E	Equateur	rainy	Back yard	Sera, Swabs, Tissues	positive	positiv	Severe clinical disease
34	Oct 2011	Lisala	4°05′N, 21°30′E	Equateur	rainy	Back yard	Sera, Swabs, Tissues	positive	positive	Severe clinical disease
35	Mar 2012	Tshela	5°20′S,13°16′ E	Bas Congo	rainy	Back yard	Sera, Swabs, Tissues	positive	positive	Severe clinical disease

Supplementary Table 2. Estimate losses in USD during PPR outbreaks in caprine in some rural and peri-urban areas of DRC, 2010-2012.

Year	Province	District	Location	Mortality	Unit price (usd)	Total (usd)
2010	Equateur	Sud Ubangi	Zongo	7312	40	292480
		Sud Ubangi	Gemena	6808	40	272320
		Sud Ubangi	Kungu	8026	35	280910
		Equateur	Bomongo	37	35	1295
			Mbandaka	117	60	7020
		Tshuapa	Boende	5229	30	156870
		Mongala	Lisala	1472	40	58880
	Bandundu	Kwilu	Gungu	519	50	25950
		Mai-Ndombe	Oshue	196	30	5880
		Mai-Ndombe	Mushie	2021	30	60630
		Plateau	Kwamouth	298	50	14900
		Kwilu	Gungu	7262	50	363100
		Kwilu	Kikwit	4257	50	212850
		Kwilu	Idiofa	4715	40	188600
		Kwilu	Bulungu	2150	40	86000
		Kwilu	Bagata	3176	40	127040
		Kwilu	Masimanimba	3962	50	198100
		Kwilu	Bandunduville	52	50	2600
		Kwango	Kenge	248	50	12400
		Kwango	Kasongo Lunda	1037	40	41480
		Kwango	Feshi	522	40	20880
	Kasaï Or.	Sankuru	Lodia	75	50	3750
		Sankuru	Lodia	24	50	1200
	Bas Congo	Lukaya	Madimba	11	50	550
		Boma	Boma	14	50	700
		Bas-Fleuve	Tshela	4636	50	231800
		Lukaya	Inkisi	289	60	16800
	Kasaï Occ.	Lulua	Ndemba	64703	50	3235150
		Lulua	Dibaya	17487	45	786915
		Lulua	Dimbelenge	35779	50	1788950
		Lulua	Kazumba	62197	40	2487880
		Lulua	Lwiza	120113	50	6005650
		Kasaï	Dekese	1765	40	70600
		Kasaï	Ilebo	19017	50	950850
		Kasaï	Luebo	71891	45	3235095
		Kasaï	Mweka	194613	50	9730650
		Kasaï	Tshikapa	28634	50	1431700
		Kasaï	Ville de Kananga	7630	70	534100
		Kasaï	Ville de Tshikapa	1931	70	135170
	Ville de Kinshasa	Lukunga	Kimwenza	37	80	2960
	Nord-Kvu	Lubero	Lubero	358	40	14320
2011	Bandundu	Kwilu	Bulungu	3696	40	147840
		Kwilu	Masimanimba	35 459	50	1772950
		Kwilu	Kikwit	921	50	46050
		Kwilu	Gungu	2481	50	124050
		Kwilu	Idiofa	545	40	21800
		Kwango	Feshi	80	40	3200
		Kwango	Kenge	2887	50	144350
		Plateau	Kwamouth	192	50	9600
	Equateur	Sud Ubangi	Gemena	2853	40	114120
		Sud Ubangi	Budiala	100	35	3500
		Sud Ubangi	Kungu	176	35	6160
		Sud Ubangi	Libenge	301	35	10535
		Sud Ubangi	Makanza	47	40	1830
		Sud Ubangi	Bikoro	924	40	36960
		Mongala	Lisala	2751	40	110040
	Bas Congo	Songololo	Songololo	67	50	3350
		Cataractes	Mbanza-Ngungu	42	60	2520
		Bas Fleuve	Boma	292	50	14600
		Ville de Matadi	Matadi	57	60	3420
